# Magnitude and associated factors of suicidal behavior among postpartum mothers attending public health centers of Addis Ababa, Ethiopia

**DOI:** 10.1186/s12888-022-04090-z

**Published:** 2022-07-13

**Authors:** Selamawit Tilahun, Berhanu Wordofa Giru, Worknesh Snshaw, Natnael Moges

**Affiliations:** 1 Addis Ababa University, Tikur Anbesa Specialized Hospital, NICU, Addis Ababa, Ethiopia; 2 Department of Psychiatry, Addis Ababa University, College of Health Science, School of Nursing And Midwifery, Addis Ababa, Ethiopia; 3 Addis Ababa University, College of Health Science, School of Nursing and Midwifery, Addis Ababa, Ethiopia; 4 Department of Pediatrics and Child Health Nursing, Debretabor University, College of Health Science, Debretabor, Ethiopia

**Keywords:** Suicidal behavior, Associated factors, Postpartum mothers, Ethiopia

## Abstract

**Background:**

In low-income nations, suicidal conduct increases within the first year following delivery, ranging from 4 to 17.6%, with a three-fold cause of maternal death. Suicidal behavior was also reported to be 14% among postpartum women in Ethiopia.

**Objective:**

To assess the magnitude and associated factors of suicidal behavior among postpartum mothers attending public health centers in Addis Ababa, Ethiopia, 2021.

**Methods:**

A cross-sectional study was conducted in ten public health facilities of Addis Ababa, Ethiopia with a total sample size of 615 women. The data were entered into Epi data 4.6 and exported to SPSS version 25 for statistical analysis. A logistic regression model with adjusted odds ratio (AOR), 95% confidence interval (CI) and *p*-value of ≤0.05 was used to identify predictors of the outcome variable.

**Results:**

The magnitude of suicidal behavior among postnatal mothers attending public health centers in Addis Ababa was 41.46% with 95%CI (35.2-44.5%). Being mother’s literate (adjusted odds ratio (AOR) = 0.64, 95% CI: 0.42-0.97), verbal abuses (AOR = 2.18, 95% CI: 1.38-3.44), history of rape (AOR = 3.03, 95% CI 1.14 -8.05), history of depression (AOR = 4.12, 95% CI 1.21-14.03), women’s having sexually unfaithful husband (AOR = 1.42, 95% CI 1.14-6.23) and khat chewing (AOR = 8.48, 95% CI 2.52-28.50) were significantly associated with suicidal behavior.

**Conclusion:**

The magnitude of suicidal behavior among postnatal mothers attending public health centers in Addis Ababa was 41.46% and it was found to be associated with being literate, rape, verbal abuse, having a history of depression, having a sexually unfaithful husband and chewing khat. As a result, women should be screened for suicidal behavior during antenatal and postnatal services for early detection and management.

## Introduction

Nearly one million people die by suicide each year; 10–20 million people consider suicide; and 50–120 million people are affected by a family member’s death or attempted suicide. Asia accounts for 60% of global suicides, implying that at least 60 million people in Asia are impacted by suicide or attempted suicide each year [1].

In women, suicidal behaviors are among the major contributors to the global burden of disease [2]. Maternal suicides occurred at a rate of one per 92,982 live births, and the annual suicide rate in women who were or had recently been pregnant varied from zero to 0.2 per 100,000, compared with 3.1-5.2 in the female population as a whole [3]. A study conducted in North Central Province found that 17.8% of recorded maternal deaths were due to suicide; ranking it number one among causes of maternal deaths [4].

In low-income countries the prevalence of suicidal behavior (SB) among mothers is different. For instance, in Tanzania prevalence was 29% lifetime suicidal thought; where in Peru province a 0.8% difference [2]. The occurrence of suicidal behavior is higher in the first year after delivery: varying from 4 to 17.6% [5–7]. It causes maternal death which is three times more in low-income countries than in developed countries [8]. Even though the prevalence of suicidal behavior is high in low-income countries like Ethiopia 14% [9], the community attitude is misrepresented. Not specific to women, suicidal behavior may be considered a sin among family members in Ethiopian culture. As a result, suicidal behavior may be associated with stigma or shame, as well as having a psychosocial impact on women who have attempted suicide [10].

Studies showed that self-reported suicidal ideation is significantly associated with an increased risk for suicide attempt or death [11]. Women having psychological disturbance during pregnancy and in the postnatal period have an increased risk for suicidal behavior [12–14]. As a result, suicidal behavior is the leading cause of death among mothers with psychopathological disorders [15, 16]. Studies that have examined postpartum suicidal behavior have found it to be associated with depression and to be the leading cause of maternal death during the postpartum year [17, 18]. In addition to other major factors which lead to suicidal behavior age [16], educational level [5], being born in low economic countries [8], domestic violence [2] marital status [19], and having a baby who is hospitalized or loosening an infant [12] were the factors associated with suicidal behavior among postpartum mothers.

In Ethiopia, little is known regarding the relationship between hormonal changes after childbearing in the post-natal period, postpartum anxiety and depression, psychological and clinical factors, and suicide conduct in women, to our knowledge. More research on suicidal behavior in women is needed, particularly in underdeveloped nations. As a result, the current study aims to determine the extent of suicide conduct and its associated characteristics among postnatal women in Addis Ababa’s public health institutions.

## Methods

### Study area and period

The study was conducted among postpartum women attending public health centers in Addis Ababa, Ethiopia from February 1 to March 15 /2021. Addis Ababa is Ethiopia’s capital city, having a diverse ethnic population that includes practically every ethnic group present in the country. Addis Ababa is divided into 11 sub-cities, each with 118 woredas, and is situated at a height of 7700 ft (2355 m). According to the population projection value for 2021, the city has an estimated total population of 5,005,524 [20]. According to Addis Ababa city health office report, the city has 101 health centers and each sub-city has 7-13 health centers.

### Study design and population

A health facility-based cross-sectional study was conducted. All postnatal mothers attending the public health centers of Addis Ababa, Ethiopia for routine postnatal care were the source population.

All mothers attending the ten health centers (general checkups for the mother and the baby including immunization which is mandatory during the 6-week postpartum) were included in the study from February 1 to March 15 /2021.Those mothers who refuse to participate and who were seriously sick were excluded from the study.

### Sample size determination

A total of 615 post-partum mothers were obtained using the single proportion formula; considering a confidence level of 95%, marginal error of 5%, a reasonable estimate of suicidal behavior from a previous study (*P* = 0.14) [21] and adding a non-response rate of 10%. Because of the multistage sampling technique; the sample size was multiplied by the design effect. By taking 3 as the design effect, the required sample size was 615.

### Sampling procedure and technique

The study’s respondents were chosen using a multistage sampling process. Using a simple random sample procedure, four sub-cities (Nifasilik lafto, Lideta,, Gulele and Kirkos) were chosen among eleven sub-cities located in the Addis Ababa municipal administration. Second, a total of 10 health centers were selected randomly from a total of 33 health centers found in the four sub-cities (2 from Nifasilik lafto and Lideta each and 3 from kirkos and Gulele each). The proportion to size allocation technique was used to calculate the number of women to be included in the study from the selected health centers based on the previous month’s data from the selected health centers. The mothers who took part in the study were chosen using systematic random sampling. The first woman was chosen by random, and after that, every two women who visited the health centers were selected to participate in the study.

## Measurements

The questionnaire was initially in English and was translated into the Amharic language (local working language). The questionnaire consists of the Socio-demographic characteristics (age, education, marital status, grew up with, job, relative wealth, and age at marriage), Psychosocial related characteristics (verbal abuse, physical abuse, history of rape, social support, substance user husband, sexually unfaithful husband, alcohol use, smoking, khat chewing) and Clinical related characteristics (parity, pregnancy, history of abortion, infant health status, history of depression) [5, 9, 22].

Suicidal behavior was measured using the suicidal screening tool which is part of the Mini-International Neuropsychiatric Interview, and it was set based on the Diagnostic and Statistical Manual of Mental Disorders-IV criteria and includes the following questions. Over the last month: Have you wished you were dead? Have you thought of committing suicide? Have you wanted to harm yourself? Have you attempted suicide? Have you ever attempted suicide? Have you planned how to commit suicide?

If the mother responds ‘yes’ to one of the six questions, she is considered at risk for suicidal behavior [23]. This tool is short and structured and has good validity and reliability in screening suicidal behavior [24].

The Oslo-3 social support scale was used to assess social support [25], which assesses the number of close confidants, the reported amount of care from others, and the perceived ease of seeking aid from neighbors. Substance use was measured using Alcohol, smoking and substance involvement screening test [26]. Abuse was evaluated by using abuse assessment scale [27], which is helpful tool to asses life time and current abuse among mothers in clinical setting .

The mothers current wealth was estimated by asking them how wealthy they thought they were in comparison to other individuals in the community [28]. They were also asked to compare their personal income to that of others, categorizing it as less than others, similar to others, or more than others.

### Operational definitions

Social support -If the mother had close confidants, sense of concern from other people and relationship with neighbours with accessibility of practical help is taken as social support.

Substance use – Recurrent use of alcohol, drug khat chewing and smoking by the mother is considered as substance user.

Abuse – If the mother is manipulated and controlled by someone emotionally, physically without her interest is considered as abuse.

Sexually unfaithful husband -To know sexually unfaithful husband, mothers are asked about her husband history of cheating and if this is true, the husband is termed sexually unfaithful.

### Data quality assurance and analysis

Data collection instruments were Pretested on 5% of non-study participants that fulfil the inclusion criteria to check the accuracy of responses, language clarity, appropriateness of data collection tools, estimate the time required and the necessary amendments were considered based on it before the actual data collection. To confirm the questions are internally consistent we validated by Cronbach’s Alpha test and pilot testing. We did Cronbach’s Alpha test for all questions and the result was 72%, indicating excellent internal consistency in the responses**.**

Five nurses and one supervisor who were not employees of the selected health centers were assigned as data collectors and they were trained for one day on information about the research objective, eligible study subjects, data collection tools and procedures, and interview methods. After data collection, filled data was entered to Epi data 4.6 and exported to SPSS version 25 statistical software for further analysis. All variables with a *p*-value ≤0.25 were taken into the multivariable logistic regression model to control for all possible confounders. A logistic regression model with adjusted odds ratio (AOR), 95% confidence interval (CI) and p-value of ≤0.05 was used to identify predictors of the outcome variable.

**“**Hosmer and Lomeshow” goodness of fit test was used to assess the overall goodness of the model with the fitted data. In addition, the receiver operating characteristic curve (ROC curve), which is produced by plotting the true positive rate against the false positive rate of the model at various thresholds, was used to check the discriminative power of the model, and an area under the curve (AUC) value of greater than 70% was used to categorize the model as it can adequately classify individuals with and without the outcomes.

### Assumption of binary logistic regression

**Fig. 1:**
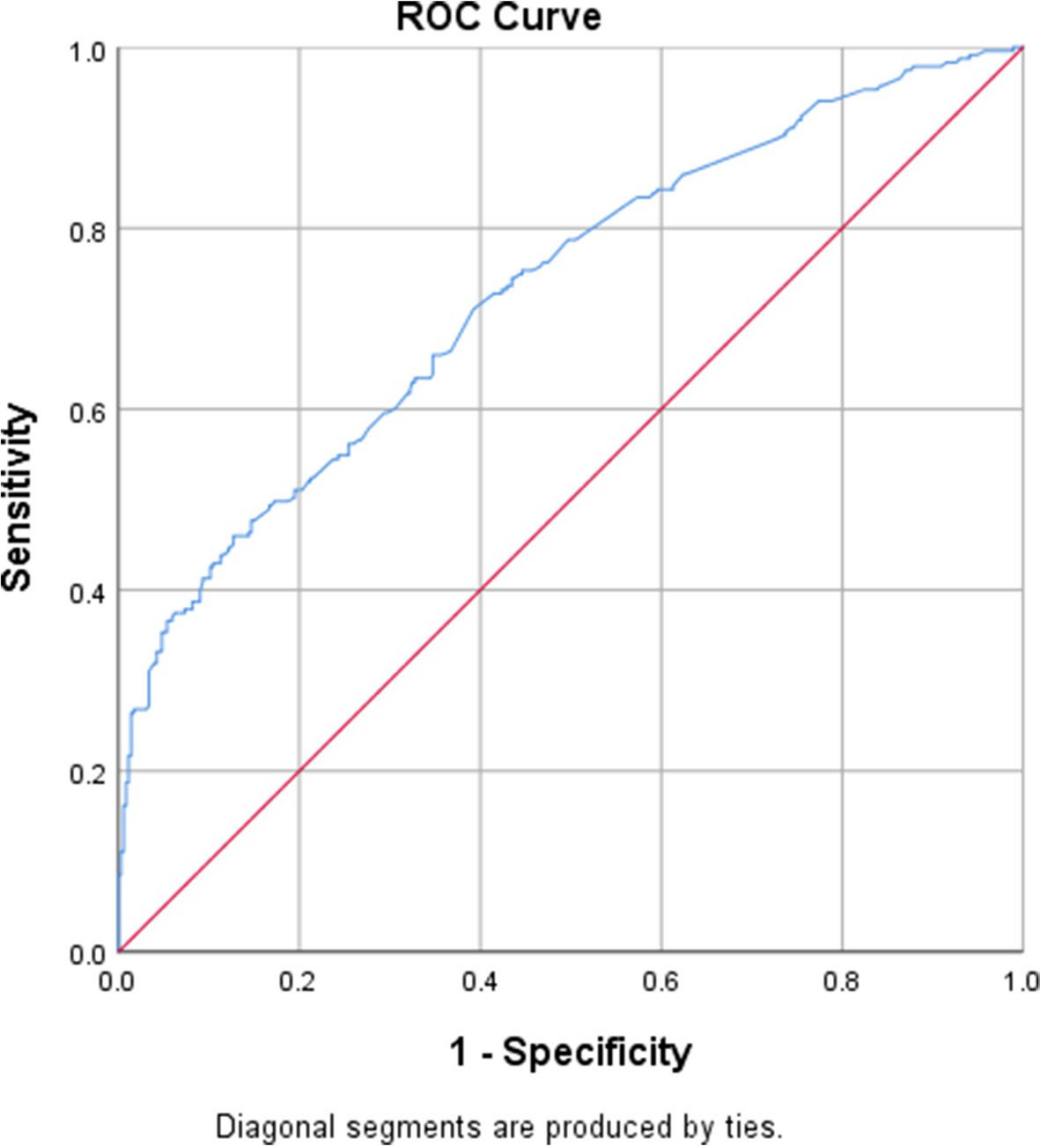
ROC curve graph showing model discrimination power towards magnitude of suicidal behavior among post-partum mothers attending at Addis Ababa public health centers, Addis Ababa, Ethiopia, 2021

Hosmer and Lomeshow goodness of test for multi logistic regression revealed that the model constituting such variables is good to predict the outcome variable i.e. magnitude of suicidal behavior (*P*-value =0.530). Indeed, the discrimination power of the model was checked by the receiver operating curve and the area under the curve value (AUC value = 0.730) illustrated that the model is excellent for discriminating individuals with Suicidal behavior (true positives) and individuals without suicidal behavior (Fig. 1).

## Result

### Socio-demographic characteristics

With a 100% response rate, 615 postpartum women were enrolled in the study. The majority of the mother 64.23% age was between 25 and 34 years. Of the total study subjects, 72.03% were literate. Three hundred forty-six (56.26%) of the respondents were jobless. Regarding marital status, 92.85% women were married. Three hundred twenty-five (54.44%) mothers married at the age ranging between 21 and 25 years (Table 1).

**Table 1:** Socio-demographic characteristics of women in postpartum period in Addis Ababa, Ethiopia, 2021 (*N* = 615)

Variables	Suicidal behavior		Frequency (*N* = 615)	Percentage (%)
	Yes	No		
Age				
18-24	46	85	131	21.3%
25-34	166	229	395	64.2%
> 34	43	46	89	14.5%
Education				
Literate	171	272	443	72.0%
Cannot read and write	84	88	172	28.0%
Grew up with				
Mother	162	239	401	65.2%
Stepmother	8	1	9	1.5%
Relatives	85	120	205	33.3%
Marital status				
Currently married	229	342	571	92.8%
Currently unmarried	26	18	44	7.2%
Job				
Has job	110	159	269	43.7%
Jobless	145	201	346	56.3%
Current wealth				
Lower	55	39	94	15.3%
Same	117	176	293	47.6%
Higher	83	145	228	37.1%
Age at marriage *n* = 597				
Younger than 20 yrs	70	121	191	32%
21-25 yrs	134	191	325	54.4%
26 yrs. and above	37	44	81	13.6%

### Psychosocial characteristics

Among all the study subjects 23.74% had experienced verbal abuse in addition 55 mothers had physical abuse from an intimate partner. Furthermore, 5.53% mothers had a history of rape once in their lifetime. Of all mothers who were found to be married 7.13% revealed they had a sexually unfaithful husband. Regarding substance use, 41 women reported that they chewed Khat, 20.98% of mothers drank alcohol (Table 2).

**Table 2:** Psychosocial characteristics among women in postpartum period in Addis Ababa, Ethiopia, 2021 (*N* = 615)

Variables	Suicidal behavior		Frequency (N-615)	Percentage
	Yes	No		
**Verbal abuse**				
Yes	89	57	146	23.7%
No	166	303	469	76.3%
**Physical abuse**				
Yes	37	18	55	8.9%
No	218	342	560	91.1%
**History of rape**				
Yes	27	7	34	5.5%
No	228	353	581	94.5%
**Social support**				
Good	39	77	116	18.9%
Poor	216	283	499	81.3%
**Substance user husband (*n*** **= 590)**				
Yes	26	9	35	5.9%
No	210	345	555	94.1%
**Sexually unfaithful husband (*n*** **= 589)**				
Yes	32	10	42	7.1%
No	203	344	547	92.9%
**Alcohol use**				
Yes	73	56	129	21%
No	182	304	486	79%
**Smoking**				
Yes	25	5	30	5%
No	230	355	585	95%
**Khat chewing**				
Yes	37	4	41	7%
No	218	356	574	93%
**Cannabis use**				
Yes	23	7	20	3.3%
No	225	360	595	96.7%

### Clinical characteristics

Two hundred forty-three (39.5%) of mothers respond it was their first pregnancy. Of all the participants 13.17% declared the current pregnancy was unplanned. Moreover, 18.70% of mothers had a history of abortion (Table 3).

**Table 3:** Clinical characteristics among women in postpartum period in Addis Ababa, Ethiopia, 2021 (*N* = 615)

Variables	Suicidal behavior		Frequency (N-615)	Percentage
	Yes	No		
**Parity**				
First	110	133	243	39.5%
Second	85	153	238	38.7%
Third	45	55	100	16.3%
Fourth	15	19	34	5.5%
**Pregnancy**				
Planned	202	332	534	86.8%
Unplanned	53	28	81	13.2%
**History of abortion**				
Yes	52	63	115	18.7%
No	203	297	500	81.3%
**Infant health status**				
Sick	20	4	24	3.9%
Healthy	235	356	591	96.1%
**History of depression**				
Yes	26	5	31	5%
No	229	355	584	95%

### Magnitude of suicidal behavior among postpartum women

Of all the study subjects enrolled in the study magnitude of suicidal behavior was 41.46%. Of these 98.45% wished they were dead, 66.93% wanted to harm themselves, 14.57% of women have planned how to commit suicide, 25% had suicidal ideation, 12.99% of the respondents had attempted suicide.

### Associated factors of suicidal behavior among post-partum mothers

All variables were undergone bivariable logistic regression and variables namely: the age of the mother, educational status, grew up with, marital status, job, current wealth, unplanned pregnancy of the current child, infant health status, physical abuse, verbal abuse, history of rape, history of depression, substance user husband, sexually unfaithful husband, alcohol use, smoking, and khat chewing were run in to further analysis.

In multivariable logistic regression analysis, educational status, verbal abuse, history of rape, history of depression, a sexually unfaithful husband, and khat chewing has significant association with suicidal behavior (Table 4).

**Table 4:** Bi-variate and multivariate logistic regression of suicidal behavior among women in the postpartum period, in health centers of four selected sub-cities of Addis Ababa, Ethiopia, 2021 (*n* = 615)

Variable	Suicidal behavior		COR (95% CI)	AOR (95%CI)
	Yes n (%)	No n (%)		
**Education status**				
Literate	171 (27.80%)	272 (44.22%)	0.66 (0.46-0.94)	0.64 (0.42-0.97) *
Cannot read and write	84 (13.65%)	88 (14.30%)	1	1
**Verbal abuse**				
Yes	89 (14.47%)	57 (9.29%)	2.85 (1.94-4.18)	2.18 (1.38-3.44) *
No	166 (26.99%	303 (49.26%)	1	1
**History of rape**				
Yes	27 (4.39%)	7 (1.13%)	5.97 (2.56-13.94)	3.03 (1.14-8.05) *
No	228 (37.07%)	353 (57.39%)	1	1
**History of depression**				
Yes	26 (4.22%)	5 (0.81%)	8.06 (3.35-21.29)	4.12 (1.21-14.03)*
No	229 (37.23%)	355 (57.7%)	1	1
**Sexually unfaithful husband** ***n*** **= 589**				
Yes	32 (5.20%)	10 (1.62%)	2.18 (1.09-7.38)	1.42 (1.14-6.23)*
No	203 (33%)	344 (55.93%)	1	1
**Khat chewing**				
Yes	37 (6%)	4 (0.65%)	15.11 (5.31-42.69)	8.48 (2.52-28.50)*
No	218 (35.44%)	356 (57.88%)	1	1

1 = Reference

*Statistically significant by AOR at *p*-value < 0.05

The odds of education status of the mother who is literates were 36% (AOR = 0.64, 95% CI: 0.42-0.97) less likely to have suicidal behavior when compared with mothers who cannot read and write.

Mothers who had Verbal abuse were 2.18 (AOR = 2.18, 95% CI: 1.38-3.44) times more likely to risk suicidal behavior compared with mothers who had no verbal abuse.

History of rape among mothers was also found to affect the outcome variable. Those women who had a history of rape were 3.03 (AOR = 3.03, 95% CI 1.14 -8.05) at higher risk for suicidal behavior compared with mothers who had no history of rape by odds of. Those women who had a history of depression were four times at higher odds of having suicidal behavior as compared to women who had no history of depression (AOR = 4.12, 95%CI1.21-14.03).

Women having sexually unfaithful husbands were 42% (AOR = 1.42, 95% CI 1.14-6.23) high likely to have suicidal behavior compared with mothers who had a sexually faithful husband.

In addition, mothers who chew khat were 8 times at higher risk for suicidal behavior compared to those who don’t chew khat (AOR = 8.48, 95% CI 2.52-28.50).

## Discussion

The finding of this study revealed the magnitude and associated factors of suicidal behavior among postnatal women who gave birth in different health centers in Addis Ababa, Ethiopia. The study participants were selected from women who came for postnatal care and vaccination services in health centers. Findings from this study might therefore highlight the current levels of suicidal behavior among postpartum women and its associated factors. In addition, it could confirm the need and possibility of integrating suicidal behavior screening into antenatal, postnatal, and child health services.

The study indicated 255 (41.46%) respondents were having suicidal behavior during their postpartum period. This result implied that a significant proportion of women were experiencing suicidal thoughts and ideation, hence maternal mental health problem is a concern enhancing for which integrated services are crucially needed. This was moderately comparable with the study which was conducted in South Africa found an equivalent prevalence rate where suicidal behavior was 41.2% [22]. Under other conditions this figure was higher when compared to other similar studies done in Brazil (11.5%) [5], Peru (12.0%) [2], United Kingdom (9.0%) [29], Serbia (1.9%) [2], Tanzania (0.8%) [2]. In a similar study conducted in Northwestern Ethiopia results implied prevalence of suicidal behavior among post-partum mothers was 14.0% [9]. A discrepancy in results might be due to the different tools, assessment period, methods, and economic status. For instance, the study in Brazil used only Three suicidal outcomes were included in this analysis to assess suicidal behavior (suicidal thoughts in the past four weeks, ever thinking about suicide, and ever attempting suicide) [2]. Furthermore, an ongoing prospective cohort study of pregnant women was used to assess suicidal behavior in a study conducted in Peru [30].

Those women’s who went to school or literate were 34% less likely to have suicidal behavior when compared with mothers who didn’t go to school or unable to read and write. This result is contrary to other similar study, which might be due to the differences in a health facility, study design, variable category, and sample size variation across studies [5].

Verbal abuse was found to have a substantial connection with suicidal conduct. Moms who experienced verbal abuse were 2.18 times (AOR = 2.18,95% CI; 1.38-3.44) more likely to have suicidal behavior than mothers who did not experience verbal abuse. Postpartum women who were verbally abused by a partner were more likely to have suicidal thoughts and attempt suicide.

Women who have been subjected to verbal abuse are at high risk to develop mental problems and physical diseases, which can lead to pregnancy-related issues such as the birth of premature children with low birth weight [31]. This could be because it is illegal for women in Ethiopian society to respond to their husbands, thus the frequent verbal abuse leads to withdrawal from life.

Another significant link discovered in the study was between moms who had been raped as children and suicidal behavior. Previous research has found that moms with a history of rape or physical abuse are more likely to have suicidal behavior and other psychiatric illnesses later in life [31]. The incidence of suicide among women who have suffered violence reveals a substantial correlation and there was a 15-fold variance in the prevalence of suicide attempts [2]. Furthermore, moms who had been diagnosed with depression as a result of their current kid were four times more likely than mothers who had not been diagnosed with depression to engage in suicide behavior [32]. This conclusion supports prior research that found a history of depression to be a risk factor for suicide behavior.

Postpartum depression was found to have the largest impact on the likelihood of suicide. When assessing the risk factors for suicide conduct, comorbidity is an important element to consider. Psychological illnesses are more common in combination with depression, which is a substantial risk factor for suicide conduct, than in isolation [5]. Suicidal behavior is found to be much greater in people suffering from postpartum depression, implying that 10-27% of persons suffering from postpartum depression will attempt suicide at least once in their lives [29].

In comparison to mothers who had a sexually faithful husband, study participants who had sexually unfaithful husbands were 42% more likely to commit suicide. This could be related to their discontent with their marriage. This is corroborated by a research conducted in the United States, which indicated that “issues with a current or former intimate relationship” were linked to suicidal behavior in 28% of women [33]. Suicide may be considered by women because they blame themselves for their husbands’ infidelity, leading to maladaptive coping mechanisms [34].

In the present study, mothers who chew khat was 8 times at higher risk for suicidal behavior compared with mothers who don’t chew khat. This finding was in line with the literature despite additional substances were also used for instance in a study conducted in the USA the category substance was used for (illicit substances, alcohol, and tobacco) [35]. Studies have previously analyzed the association between alcohol use during pregnancy and suicidal behavior; both reporting a significant positive association, one exploring factor associated with self-poisoning and current suicide risk [7]. Therefore, addictive behavior during pregnancy and post-partum periods will aggravate existing mental health problems and negative birth outcomes resulting in post-partum depression adding to suicidal behavior.

### Strength and limitations of the study

The study’s strength was analyzing the suicidal behavior of mothers who may be suffering from a variety of psychosocial pressures in low-income setting. The first flaw in this study was that teenage moms, who may report frequent suicidal behavior, were left out, because of exaggerating the scale of suicide behavior. Second, depression was not screened in this study, and only known cases of depression were included. Third, the research design makes it difficult for the researcher to determine cause and effect.

## Conclusion

The magnitude of suicidal behavior among postnatal mothers attending public health centers in Addis Ababa was 41.46% and it was found to be associated with being literate, rape, verbal abuse, having a history of depression, having a sexually unfaithful husband and chewing khat. As a result, women should be screened for suicidal behavior during antenatal and postnatal services for early detection and management.

## Data Availability

The data are accessible from the corresponding author upon reasonable request.

## Abbreviations

TASH: Tikur Anbesa Specialized Hospital
NICU: Neonatal Intensive Care Unit
AOR: Adjusted odds ratio
CI: Confidence Interval
SB: Suicidal behavior
SPSS: Statistical package for social science
